# Prolactin measurements in a real-life setting: a population-based cohort study

**DOI:** 10.1007/s11102-026-01647-z

**Published:** 2026-03-25

**Authors:** Christoffer Krogager, Jens Otto Lunde Jørgensen, Claus Højbjerg Gravholt, Kirstine Stochholm

**Affiliations:** 1 Department of Internal Medicine, Regional Hospital Horsens, Horsens, Denmark; 2 Department of Endocrinology, Aarhus University Hospital, Aarhus, Denmark; 3 Department of Clinical Medicine, Aarhus University, Aarhus, Denmark; 4 Department of Molecular Medicine, Aarhus University Hospital, Aarhus, Denmark; 5 Krogagre 58, Risskov, DK-8240 Denmark

**Keywords:** Prolactin, Hyperprolactinemia

## Abstract

**Purpose:**

Hyperprolactinemia is mainly due to pituitary disorders including prolactinomas but other causes including adverse drug reactions exist. This study aimed to assess the frequency and pattern of serum prolactin measurements in a population-wide cohort in Central Region Denmark (1.32 mill inhabitants) from 2011 to 2022.

**Methods:**

Using the Central Denmark Region’s data warehouse, we identified all individuals who had at least one prolactin measurement in the study period.

**Results:**

We identified 84,145 individuals who had at least one measurement of prolactin. Among these, 17,008 (20.3%) had at least one measurement of elevated prolactin corresponding to an incidence of 107 per 100.000 person-years. Hyperprolactinemia, defined by at least two elevated prolactin concentrations, occurred in 5,810 individuals (73.9% female), with an incidence of 36.7 per 100.000 person-years. Only 12.7% of individuals with hyperprolactinemia were treated with dopamine agonists.

**Conclusion:**

We found a substantial number of repeated prolactin measurements, many of which did not seem to lead to clinical action. The findings support the need for guidelines for management of hyperprolactinemia to ensure proper utilization of health resources.

## Introduction

 Prolactin is an anterior pituitary gland hormone, which is predominantly negatively regulated by hypothalamic dopamine acting on the D2 receptor on the lactotroph cells [1, 2]. The primary role of prolactin is to induce and sustain lactation during pregnancy and postpartum [3]. Non-puerperal hyperprolactinemia can be attributed to various causes, such as prolactinomas (usually benign neuroendocrine tumors arising from the pituitary gland), adverse reactions due to the use of drugs with anti-dopaminergic properties (in particular antipsychotics and antidepressants), or obstruction of the hypothalamic-pituitary dopaminergic pathway in the pituitary stalk by intracranial tumors [4]. Hyperprolactinemia can result in various symptoms, including menstrual disturbances and galactorrhea in women and hypogonadism in both females and males [4]. Where indicated, medical treatment with a dopamine agonist is first choice treatment and some patients with prolactinomas are eligible for pituitary surgery [4].

The incidence of hyperprolactinemia is estimated to be approximately 5 per 100.000 person years in a Dutch study based on patients receiving medical treatment for hyperprolactinemia [5]. A similar incidence has been found in a Danish study [6] but the incidence estimates of hyperprolactinemia vary greatly [4]. No information is available regarding the frequency and purpose of prolactin measurements in a population-wide cohort population.

Optimal health care utilization is of obvious interest and one strategy to accommodate this is to audit the usage of diagnostic testing, including blood sampling. In general practice, fertile women often describe symptoms related to polycystic ovarian syndrome (PCOS), including menstrual irregularities, or infertility, where guidelines recommend routine measurement of prolactin. Similarly, in men, symptoms of hypogonadism or infertility may lead to measurement of prolactin. In endocrinology, prolactin is frequently measured repeatedly for the differential diagnosis of women with verified menstrual irregularities as well as hypogonadism and pituitary disorders in patients of both sexes. However, the utilization of this prolactin measurement in general practice and in a hospital setting is inadequately documented. The aim of this study was therefore to quantify prolactin measurements in an unselected population-wide cohort, including individuals only in general practice, to describe the characteristics of individuals undergoing testing, and to examine the use of dopamine agonist treatment among those with elevated prolactin levels.

## Materials and methods

### Study population

The study was a population-based cohort study including all individuals living in Central Region Denmark (total population: 1.32 mill. inhabitants in 2022, corresponding to 22% of the Danish population), who had prolactin measured at least once between January 1st 2011 and December 31st 2022.

## Identification of individuals with a registration of measurement of prolactin

In Central Denmark Region, all health care data are stored in a data warehouse called the Business Intelligence (BI)-portal. Using the BI-portal, we identified the study population consisting of all individuals with at least one measurement of prolactin value during the study period, using the Nomenclature for Properties and Units (NPU) [7] code for “prolactin” ( NPU18247). We identified who requested the blood test and categorized them into four groups (general practitioner, department at a hospital excluding psychiatric departments, psychiatric department at a hospital, and outside Central Denmark Region), these groups cover the structure of the Danish healthcare system as very few private medical practices exist. Moreover, within the study population we identified all individuals who had had one or more of the following: a pituitary MRI or prescribed medication for a psychiatric disorder or dopamine agonist treatment. We defined treatment for a psychiatric disorder as medication with one of in the following Anatomical Therapeutic Chemical (ATC) classification codes [8]: N05A, antipsychotics, and N06A, psychoanaleptics. Dopamine agonist treatment (DA treatment) was defined by the following ATC codes: G02CB03, cabergolin, G02CB04, quinagolid, or N04BC01, bromocriptin.

### Identification of individuals with a normal prolactin level

We defined individuals with a normal prolactin level as individuals where all measured prolactin levels were below the age and sex specific upper reference values, and who did not have a registration of dopamine agonist treatment for a period of ≥ 30 days at any time. We chose a treatment period of a month of less as a cutoff in an attempt to ensure, that women treated with dopamine agonist for cessation of nursing were not classified as hyperprolactinemia being treated with dopamine agonists in the scope of our study. The upper reference values (international milli units/l = mU/l) used for individuals below 18 years were for females: 1343 age 0–4 years and 480 age 4–18 years, and for males 1559 age 0–4 years and 402 age 4–18 years. Above 18 years of age, the upper reference value was 580 for females and 460 for males. The cutoff values used represents the normal range of prolactin used by the central laboratory at Aarhus University Hospital [9].

### Identification of individuals with a registration of increased prolactin

We defined all individuals with a registration of increased prolactin as individuals who had at least one measurement of prolactin above the age and sex specific upper reference value, regardless of numbers of normal prolactin levels measured.

### Identification of individuals with hyperprolactinemia

We defined individuals with hyperprolactinemia as individuals who had at least two measurements of prolactin above the age and sex specific upper reference value.

### Statistical analysis

Summary statistics were applied to describe the population. Data are presented as mean +/- standard deviation (SD) or as medians with interquartile range (IQR). The incidences of increased prolactin and of hyperprolactinemia were calculated as $\left(\frac{numberofaffectedindividuals}{totalpopulation*12years}\right)*100.000$, the incidence is stated as cases/100.000 person years. Differences between groups in continuous variables were tested using a non-paired t-test. Differences between groups in categorical variables were tested using a c^2^-test. For test of differences in medians, a Wilcoxon rank-sum test was used. For all statistical tests, a p-value below 0.05 were considered statistically significant. Statistical analyses were performed using STATA 14.2 (StataCorp 4905 Lakeway Dr College Station, TX 77845, USA).

## Results

Table 1 shows baseline date on all individuals with at least one measurement of prolactin, Table 2 shows data on all with hyperprolactinemia, and Table 3 shows data on all treated with DA agonists.

**Table 1:** Data on all individuals with at least one measurement of prolactin

	TOTAL	Baseline data at firstmeasurement	Individuals with only1 measurement	Individuals with2 measurements	Individuals with 3-5measurements	Individuals with 6-10measurements	Individuals with 10 + measurements
Measurements, n	157313	84145	57318	28710	30629	17792	22864
Individuals, n	84145	84145	57318	14355	8657	2387	1428
Female n(%)	117966 (75.0)	65057 (77.3)	44333 (77.4)	11566 (80.6)	6663 (77.0)	1545 (64.7)	950 (66.5)
Age, median (IQR)	33.8 (25.6–46.7)	32.3 (24.5–44.3)	33.1 (25.0-45.6)	31.2 (25.0-40.8)	31.4 (24.4–41.9)	36.6(25.8–53.0)	43.0 (31.4–56.9)
Prolactin value (mU/l), median (IQR)	273 (181–442)	256 (178–380)	244 (173–347)	262 (183–396)	289 (191–484)	331 (190–666)	403 (190–896)
Measurements with increased prolactin value, n(%)	36733 (23.4)	13699 (16.3)	2708 (4.7)	5185 (18.1)	8352 (26.9)	6731 (37.8)	10500 (45.9)
MRI, n (%)	3610 (4.3)	-	676 (1.2)	499 (3.5)	823 (9.5)	764 (32.0)	848 (59.4)
Individuals receiving psychiatric medication, n (%)	12970 (15.4)	-	7406 (12.9)	2390 (16.7)	2089 (24.13)	783 (32.8)	303 (21.2)
Individuals receiving treatment with dopamine agonists, n (%)	804 (0.96)	-	12 (0.02)	16 (0.11)	85 (0.98)	226(9.5)	465 (32.6)
Place of test request: Hospital in Central Region Denmark/Psychiatric department/General practitioner/Outside Central region Denmark	63746 (40.5)10903 (6.9)78374 (49.8)4290 (7.7)	24600 (29.2%)3919 (4.7%)54098 (64.3%)1528 (1.8%)	16961 (29.6)2016 (3.5)37713 (65.8)628 (1.1)	8221 (28.6)1470 (5.1)18544 (64.6)475 (1.7)	10639 (34.7)3189(10.4)15672 (51.2)1129 (3.7)	10215 (57.4)2605 (14.6)3779 (21.2)1193 (6.7)	17710 (77.5)1623 (7.1)2666 (11.7)865 (3.8)
Number of measurements pr individual, median (IQR)	1 (1–2)	1 (1–1)	1 (1–1)	2 (2–2)	3 (3–4)	7 (6–9)	14 (12–18)
Number of individuals with at least one increased prolactin value, n(%)*	17008 (20.3)	136999 (16.3)	6065 (10.6)	4101 (28.6)	4138 (47.8)	1611 (67.5)	1093 (76.5)
Number of increased prolactin values pr individual, median(IQR)	1 (1–2)	1 (1–1)	1 (1–1)	1 (1–2)	2 (1–3)	4 (2–6)	9 (5–13)

*IQR* interquartile range, *mU/l* international milli units/l. * For definition of ‘at least one increased prolactin value’, see Materials and methods

**Table 2:** Data on individuals with at least two increased prolactin values (hyperprolactinemia)

	TOTAL	Baseline data at firstmeasurement	Individuals with2 measurements	Individuals with 3-5measurements	Individuals with 6-10measurements	Individuals with 10 + measurements
Measurements, n	38147	5810	2168	8956	10123	16900
Individuals, n	5810	5810	1084	2384	1334	1008
Female n (%)	4294 (73.9)	4294 (73.9)	818 (75.5)	1791 (75.1)	939 (70.4)	746 (74.0)
Age, median (IQR)	35.4 (25.8–48.2)	30.7 (23.2–43.5)	31.0 (24.2–44.3)	29.8 (23.2–41.3)	34.1 (48.0)	39.9 (29.5–51.4)
Prolactin value (mU/l), median (IQR)	606 (363–1017)	713 (498–1132)	661 (546–960)	607 (425–901)	583 (340–983)	602 (308–1135)
Measurements with increased prolactin value, n (%)	25535 (66.9)	4646 (80.0)	2168 (100)	6498 (72.6)	6454 (63.8)	10415 (61.6)
MRI, n (%)	1608 (27.7)	-	71 (6.6)	352 (14.8)	508 (38.1)	677 (67.2)
Individuals receiving psychiatric medication, n (%)	1760 (30.3)	-	262 (24.2)	734 (30.8)	515 (38.8)	247 (24.5)
Individuals receiving treatment with dopamine agonists, n (%)	736 (12.7)	-	8 (0.7)	74 (3.1)	210 (15.7)	444 (44.1)
Place of test request: Hospital in Central Region Denmark/Psychiatric department/General practitioner/Outside Central region Denmark	21791 (57.1)4735 (12.4)9360 (24.4)2261 (5.9)	1847 (31.8)746 (12.8)2791 (48.0)426 (7.3)	739 (34.1)246 (11.4)1094 (50.5)89 (4.1)	3245 (36.2)1441 (16.1)3711 (41.4)559 (6.2)	5109 (50.5)1870 (18.5)2273 (22.5)871 (8.6)	12698 (75.1)1178 (7.0)2282 (13.5)742 (4.4)
Number of measurements pr individual, median (IQR)	4 (3–8)	1 (1–1)	2 (2–2)	4 (3–4)	7 (6–9)	15 (12–20)
Number of individuals with at least one increased prolactin value, n (%)*	5810 (100)	5810 (100)	1084 (100)	2384 (100)	1334 (100)	1008 (100)
Number of increased prolactin values pr individual, median (IQR)	3 (2–5)	1 (1–1)	2 (2–2)	3 (2–3)	5 (3–6)	10 (6–13)

*IQR* interquartile range, *mU/l* international milli units/l. * For definition of ‘at least one increased prolactin value’, see Materials and methods

**Table 3:** Data on all individuals receiving treatment with dopamine agonists

	TOTAL	First measurement	Individuals with only1 measurement	Individuals with2 measurements	Individuals with 3-5measurements	Individuals with 6-10measurements	Individuals with 10 + measurements
Measurements, n	10880	804	12	32	353	1842	8641
Individuals, n	804	804	12	16	85	226	465
Female n (%)	633 (78.7)	633 (78.7)	11 (91.7)	12 (75.0)	70 (82.4)	175 (77.4)	365 (78.5)
Age, median (IQR)	38.3 (29.5–47.6)	34.8 (27.0-43.8)	38.6 (31.6–41.8)	35.6 (28.0-49.6)	31.9 (24.8–42.1)	35.9 (28.6–45.9)	39.1 (30.0-48.1)
Prolactin value (mU/l), median (IQR)	632 (225–1235)	1170 (700.5-2358.5)	867 (256.5–1015)	751.5 (38-14065)	837 (356–1625)	649 (225–1238)	617 (259–1220)
Measurements with increased prolactin value, n (%)	6470 (59.5)	678 (84.3)	8 (66.7)	19 (59.4)	245 (69.4)	1080 (58.6)	5118 (59.2)
MRI, n (%)	630 (78.4)	-	1 (8.3)	6 (37.5)	49 (57.7)	170 (75.2)	404 (86.9)
Individuals receiving psychiatric medication, n (%)	98 (12.2)	-	0 (0)	0 (0)	9 (10.6)	31 (13.7)	58 (12.5)
Place of test request: Hospital in Central Region Denmark/Psychiatric department/General practitioner/Outside Central region Denmark	9121 (83.8)70 (0.6)1618 (14.9)71 (0.7)	345 (42.9)7 (0.9)441 (54.9)11 (1.4)	8 (66.7)0 (0)3 (25.0)1 (8.3)	23 (71.9)0 (0)6 (18.8)3 (9.4)	261 (73.9)2 (0.6)85 (24.1)5 (1.4)	1406 (76.3)0 (0)430 (23.3)6 (0.3)	7423 (85.9)68 (0.8)1094 (12.7)56 (0.7)
Number of measurements pr individual, median (IQR)	12 (8–18)	1	1	2 (2–2)	4 (4–5)	8 (7–9)	16 (14–22)
Number of individuals with at least one increased prolactin value, n (%)*	777 (96.6)	678 (84.3)	8 (66.7)	16 (100)	80 (94.1)	221 (97.8)	457 (98.3)
Number of increased prolactin values pr individual, median (IQR)	6 (4–11)	1 (1–1)	1 (1–1)	2 (1–2)	3 (2–4)	5 (3–6)	10(6–15)

*IQR* Interquartile range, *mU/l* international milli units/l. * For definition of ‘at least one increased prolactin value’, see Materials and methods

During the study period, we identified 157,313 measurements of prolactin in 84,145 individuals (75% females), corresponding to 6.4% of the total population (Table 1). We observed an increase in prolactin measurements in the first four years from 8689 in 2011 to 14,016 in 2015, thereafter the number om measurements remained stable. The increase in measurements was uniformly distributes between specialties and no clear pattern was observed. Almost half (49.8%) of the measurements of prolactin were undertaken in general practice (Table 1).

The median age of the individuals at the time of the first prolactin measurement was 33.8 years (IQR 25.6–46.7), and the age of females was significantly lower than of males (32.3 years (IQR (25.4–42.6) vs. 43.4 years (IQR 26.4–61.0), *p* < 0.001). Most of the individuals in the study (68.1%) only had a single measurement of prolactin. Only when the number of repeated measurements exceeded five were most tests ordered by a hospital department. However, even among individuals with more than ten measurements, 11.7% were ordered by a general practitioner (Table 1).

A measurement of at least one prolactin above the upper reference value was registered 36,733 (23.4%) times, representing 17.008 (20.3%) individuals of whom 12.802 (75.3%) were females (Table 1). This corresponds to an incidence of 107 per 100.000 person years (81 per 100.000 person years for females). 11.196 of the persons with elevated prolactin (80.9%) only had one elevated measurement and 6.065 of the persons with elevated prolactin had only one measurement in total. In 8.787 measurements, a normal prolactin level was observed following an elevated level in as many persons.

The total number of individuals who only had normal measurements of prolactin was 67,137 (79.8%) accounting for 97,700 tests and corresponding to 62.1% of all the tests performed.

Of the individuals where all prolactin levels were below upper reference value, 51.253 had a single measurement performed, 10.254 had two tests, 2.987 had three tests, 1.046 had four tests and 486 individuals had five tests performed, and 1,111 individuals had more than five measurements performed.

**Fig. 1:**
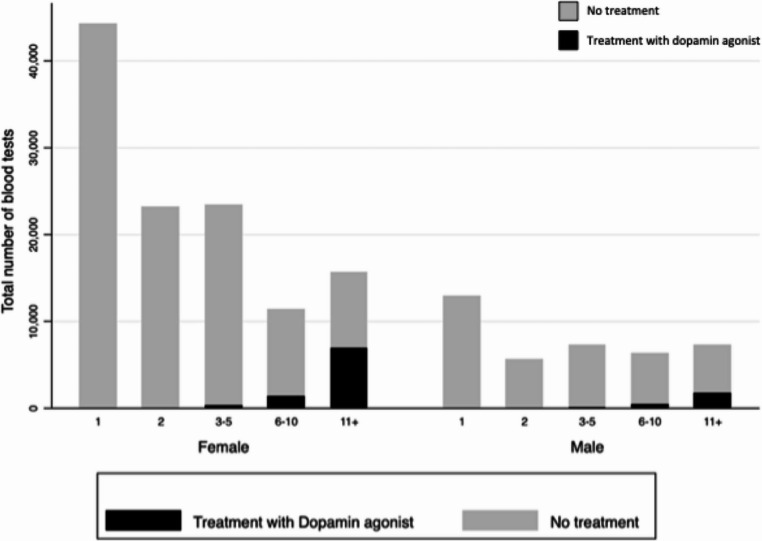
The figure shows the distribution of prolactin measurements among males and females with and without treatment with dopamine agonist treatment

The distribution of prolactin measurements and DA treatment is shown in Fig. 1.

At least two measurements of prolactin above reference value were identified in 5,810 individuals (73.9% females), corresponding to an incidence of hyperprolactinemia of 36.7 per 100.000 person years (26.6 per 100.000 person years for females) (Table 2).

Out of the total study population, 804 individuals (0.96%), corresponding to 1 out of 105, had a registration of DA treatment (Table 3). Of the 804 individuals treated with DA, 736 (91.5%) had hyperprolactinemia. Thus, among those with hyperprolactinemia (*n* = 5,810), 736 received DA treatment, corresponding to 1 out of 8. The frequency of DA treatment increased with increasing levels of prolactin, thus 1:7 with a prolactin level above 1000 mU/l received DA treatment. For prolactin levels above 1500, 2000 and 3000 mU/l, the corresponding ratios were 1:5, 1:4 and 1:3 respectively. In individuals with hyperprolactinemia, those receiving DA treatment were older, had higher prolactin levels at baseline, had a higher number of prolactin measurements and were more likely to have had a pituitary MRI performed. compared to those not receiving DA treatment (Table 4).

**Table 4:** For comparison, data on individuals where all levels of prolactin were normal and individuals who only had one elevated prolactin level are also presented

Variable	With DA treatment	Without DA treatment	*p*-value*	Normal prolactin	Only 1 elevated prolactin	*p*-value*
Age at first measurement (years, mean)	37.1	35.1	0.002	36.1	35.3	0.002
Prolactin level at first measurement (mU/l), median	4987.3	959.2	< 0.001	239	688.8	< 0.001
Number of prolactin measurements (mean)	8.7	3.8	< 0.001	1.5	1.9	< 0.001
Proportion of females (%)	79.2	73.1	< 0.001	73.7	76.0	< 0.001
Proportion of individuals with a pituitary MRI (%)	81.3	19.9	< 0.001	6.14	3.2	< 0.001
Proportion of individuals receiving antidepressant or antipsychotic medication	12.6	32.8	< 0.001	14.8	20.5	< 0.001
Days between first and second measurement, median (IQR)	30 (13–73)	48 (17–168)	< 0.001	351 (99–925)	135 (32–582)	< 0.001

*Comparison between individuals with hyperprolactinemia with and without DA treatment

*IQR* interquartile range, *mU/l* international milli units/l

Of the persons included in the study, 8144 were below 18 years of age. They accounted for 12.047 measurements equal to 7.7% of the total measurements. The patters of who ordered the tests differed from the adult population with the majority of tests being ordered by hospital departments 46.6% and psychiatry departments 17.7% and fewer tests ordered by general practitioners 35.3%. The young population had a slightly lower use of psychiatric medication 13.0% compared to the total population, data not shown.

## Discussion

Hyperprolactinemia is the most common pituitary hormonal disorder [10] although it is still considered a rare condition. With the current incidence estimate of 5 per 100.000 person years, we could expect approximately 792 individuals to develop hyperprolactinemia in the study period. However, we found more than 7 times that number of individuals with hyperprolactinemia and more than 100 times that number of individuals have had a measurement of prolactin performed.

The current recommendations form the Endocrine Society is to measure prolactin if hyperprolactinemia is suspected [4], but indications other than suspected hyperprolactinemia may prompt a prolactin measurement, e.g. routine follow-up of patients with pituitary disorders including pituitary mass lesions and hypopituitarism. A common complaint presented in general practice is menstrual disturbances which has a prevalence of 20.7% in a group of fertile young women [11]. The most common cause of oligomenorrhea and secondary amenorrhea is polycystic ovarian syndrome (PCOS) [12] and another frequent cause is premature ovarian insufficiency (POI) [13]. Endocrine Society and the European Society of Human Reproduction and Embryology, recommends measuring prolactin in females with oligo- or amenorrhea or when suspecting PCOS or POI [14].

Moreover, prolactin may be more likely to be measured in individuals receiving treatment for a psychiatric disorder [15, 16] as increased prolactin is a well-known adverse effect of several anti-psychiatric and anti-depressive drugs [17, 18]. Several other types of medication e.g. antiemetics also have the potential to elevate prolactin levels. However, the use of these medications is probably less likely to warrant a measurement of prolactin as the use of medication for psychiatric disorders.

We found a surprisingly high number of prolactin measurements in our population of whom the majority (68.1%) only had a single measurement, which accounted for 36.5% of all the measurements. Thus, these individuals probably had symptoms indicating hyperprolactinemia, which was ruled out by a normal blood test, as recommended by current guidelines [4].

A large proportion of those tested exhibited repeatedly normal prolactin levels, accounting for 41.1% of all the measurements in our study. This is a considerable number of tests, which suggests over usage that may relate to either prolactin measurement being part of a routine work up program or the result of so-called defensive medicine, which is prevalent according to both Danish and international studies [19, 20].

On the other hand, a surprisingly large number of persons, 35.7%, with elevated prolactin never had a follow-up measurement.

To our knowledge, no previous studies have prospectively detailed the pattern of prolactin measurements in a large population-wide cohort. It was expected that a large proportion of the tests were ordered by a general practitioner, since they are gate keepers and provide primary care also for premenopausal women with menstrual irregularities, which frequently triggers prolactin assessment [21]. The reason for the large number of repeated tests performed by the general practitioners, however, is unexplained and merits auditing.

The cost of prolactin tests in the study amounts to approximately 590.000€. Given the fact, that the Danish healthcare system is publicly funded and health expenditure amounts to 10.8% of the gross domestic product (GDP) [22], cost-effectiveness is a constant concern. Our price estimate is only for measuring prolactin per se, and the total cost of ordering a prolactin measurement (and likely a number of other tests at the same time) is probably much higher when accounting for e.g. the derived expenses for follow up visits, MRI, referrals to specialized medical departments. Furthermore, in many individuals, the detection of hyperprolactinemia was not followed by prolactin lowering therapy. There may be several reasons not to initiate treatment in persons with elevated prolactin e.g. lack of knowledge about the disorder in general practice, lack of symptoms in eugonadal individuals or individuals where the cause of hyperprolactinemia is suspected to be caused by treatment for a psychiatric disorder or other medications like antiemetics. Lack of treatment with DA could also be a patient preference or be owing to a different treatment strategy e.g. estrogen supplements in females with no fertility wish. If treatment is not initiated, regardless of the reason, the relevance and benefit of repeated measurements in persons with established hyperprolactinemia is questionable.

We found a surprisingly high number of patients with elevated prolactin in the study (20.3%), compared to what has previously been reported [5, 6]. However, previous data are derived from individuals receiving treatment with dopamine agonists, which likely underestimated the number of individuals with hyperprolactinemia. We chose to include the category “elevated prolactin” in our study to report the number of high measurements. Prolactin measurement is highly sensitive and an elevated prolactin level is not necessarily a pathological finding. Prolactin can be transitorily elevated for a number of reasons like medication, physical activity and sexual intercourse. The transitory nature can be seen by the fact that more than 80% of persons with elevated prolactin only had a single elevated measurement. This is also the reason we used the more stringent categorization for hyperprolactinemia requiring at least two elevated prolactin measurements.

Our study finds a likely overutilization of prolactin measurements. This may be owing to a combination of a generous screening strategy and the lack of knowledge of the effects of having mildly elevated levels of prolactin without relevant symptoms. Further studies evaluating the effects of elevated prolactin in asymptomatic individuals, on e.g. Fertility, morbidity and socioeconomics, are needed in order to gain more knowledge. This will enable the possibility for better recommendations regarding the measurement of prolactin, including when not to measure prolactin, when to initiate treatment and how to monitor these individuals.

Our study has several strengths. It includes data on a large population, with many measurements. Furthermore, the use of registry data enabled us to link use of medication and information of MRI to the prolactin data. The data in our study are real life and thus representative of current clinical practice.

Our study also has some weaknesses. The use of registry data has an inherent risk of error due to poor reporting. This risk is low in this study since the BI-portal automatically transfers data from the laboratory, and thus is not dependent on reporting. However, the fact that we set a fixed study period may result in a slight inaccuracy in our results due to the timing of measurement and initiation of treatment. For instance, more people (*n* = 804) received treatment than were classified as having hyperprolactinemia (*n* = 736) which may suggest that a few individuals started treatment before the start of our study or have moved to Central Denmark Region on dopamine treatment. Likewise, some individuals may have had an elevated prolactin measurement at the end of our study period but no record of treatment leading to misclassification of these individuals. In the same way our chosen cutoff of DA treatment for less than 30 days might have resulted in misclassification of a few individuals who may have started treatment within a month of the end of the sampling period. However, due to the long study period, we consider this a negligible risk of misclassification.

We included only the use of medication used to treat psychiatric disorders. It is well known that several other types of medication can affect prolactin levels. The lack of data on all medications used by the persons in the study is a limitation for the understanding of our findings. However, we do not believe that additional information on medicine use would alter our findings in any significant way.

Using data from registries entails a lack of granulated clinical data. Thus, we have no data on possible symptoms or the clinical reasoning for ordering blood tests. This limitation hampers a deeper interpretation of our data in a clinically significantly way.

The number of prolactin measurements was higher than anticipated, but the number of elevated prolactin levels was also quite high. Still, the number of patients in whom an elevated prolactin level led to dopamine agonist treatment remained relatively low.

Further studies examining the use of prolactin in a clinical setting as well as the clinical outcome of elevated prolactin levels are needed in order to provide future guidelines for the rationale of measuring prolactin.

## Data Availability

The raw registry data supporting the findings of this study are available from the corresponding author upon reasonable request.

## References

[1] Freeman ME et al (2000) Prolactin: structure, function, and regulation of secretion. Physiol Rev 80(4):1523–1631 11015620 10.1152/physrev.2000.80.4.1523

[2] Melmed S (2003) Mechanisms for pituitary tumorigenesis: the plastic pituitary. J Clin Invest 112(11):1603–1618 14660734 10.1172/JCI20401 PMC281651

[3] Lopez-Vicchi F et al (2020) Metabolic functions of prolactin: Physiological and pathological aspects. J Neuroendocrinol 32(11):e12888 33463813 10.1111/jne.12888

[4] Petersenn S et al (2023) Diagnosis and management of prolactin-secreting pituitary adenomas: a Pituitary Society international Consensus Statement. Nat Rev Endocrinol 19(12):722–740 37670148 10.1038/s41574-023-00886-5

[5] Kars M et al (2009) Estimated age- and sex-specific incidence and prevalence of dopamine agonist-treated hyperprolactinemia. J Clin Endocrinol Metab 94(8):2729–2734 19491225 10.1210/jc.2009-0177

[6] Steffensen C et al (2012) Heart valve disease among patients with hyperprolactinemia: a nationwide population-based cohort study. J Clin Endocrinol Metab 97(5):1629–1634 22419729 10.1210/jc.2011-3257

[7] Férard G, Dybkaer R (2013) Recommendations for clinical laboratory science reports regarding properties, units, and symbols: the NPU format. Clin Chem Lab Med 51(5):959–966 23314546 10.1515/cclm-2012-0769

[8] The selection and use of essential medicines (2006) Report of the WHO expert committee, (2005) (including the 14th model list of essential medicines). World Health Organ Tech Rep Ser (933):1-119. back cover 17566509

[9] https://www.fagperson.auh.dk/afdelinger/blodprover-og-biokemi/analysefortegnelsen/hospital/#1118067

[10] Fernandez A, Karavitaki N, Wass JA (2010) Prevalence of pituitary adenomas: a community-based, cross-sectional study in Banbury (Oxfordshire, UK). Clin Endocrinol (Oxf) 72(3):377–382 19650784 10.1111/j.1365-2265.2009.03667.x

[11] Odongo E et al (2023) Prevalence and effects of menstrual disorders on quality of life of female undergraduate students in Makerere University College of health sciences, a cross sectional survey. BMC Womens Health 23(1):152 36997915 10.1186/s12905-023-02290-7 PMC10064702

[12] Phylactou M et al (2021) Clinical and biochemical discriminants between functional hypothalamic amenorrhoea (FHA) and polycystic ovary syndrome (PCOS). Clin Endocrinol (Oxf) 95(2):239–252 33354766 10.1111/cen.14402 PMC11497304

[13] Beck-Peccoz P, Persani L (2006) Premature ovarian failure. Orphanet J Rare Dis 1:9 16722528 10.1186/1750-1172-1-9 PMC1502130

[14] Teede HJ et al (2023) Recommendations from the 2023 international evidence-based guideline for the assessment and management of polycystic ovary syndrome. Eur J Endocrinol 189(2):G43–g64 37580861 10.1093/ejendo/lvad096

[15] Jones AS et al (2021) Prolactin ordering patterns in psychiatric inpatients and the impact this has on patient management. Australas Psychiatry 29(3):282–285 32586112 10.1177/1039856220934326

[16] Walters J, Jones I (2008) Clinical questions and uncertainty–prolactin measurement in patients with schizophrenia and bipolar disorder. J Psychopharmacol 22(2 Suppl):82–89 18477624 10.1177/0269881107086516

[17] Pollock A, McLaren EH (1998) Serum prolactin concentration in patients taking neuroleptic drugs. Clin Endocrinol (Oxf) 49(4):513–516 9876350 10.1046/j.1365-2265.1998.00569.x

[18] Kearns AE et al (2000) Risperidone-associated hyperprolactinemia. Endocr Pract 6(6):425–429 11155212 10.4158/EP.6.6.425

[19] Andersen MK et al (2021) Defensive medicine in Danish general practice. Types of defensive actions and reasons for practicing defensively. Scand J Prim Health Care 39(4):413–418 34463601 10.1080/02813432.2021.1970945 PMC8725848

[20] Sekhar MS, Vyas N (2013) Defensive medicine: a bane to healthcare. Ann Med Health Sci Res 3(2):295–296 23919211 10.4103/2141-9248.113688 PMC3728884

[21] https://www.dsam.dk/vejledninger/bloedningsforstyrrelser/bilag#1-hormonanalyser-i-relation-til-bloedningsforstyrrelser. [cited 2024 3008].

[22] Birk HO et al (2024) Denmark: health system review. Health Syst Transit 26(1):1–186 38841877

